# Single-Stranded DNA Viruses in Antarctic Cryoconite Holes

**DOI:** 10.3390/v11111022

**Published:** 2019-11-04

**Authors:** Pacifica Sommers, Rafaela S. Fontenele, Tayele Kringen, Simona Kraberger, Dorota L. Porazinska, John L. Darcy, Steven K. Schmidt, Arvind Varsani

**Affiliations:** 1 Ecology and Evolutionary Biology, University of Colorado Boulder, Boulder, CO 80309, USA; steve.schmidt@colorado.edu; 2 The Biodesign Center for Fundamental and Applied Microbiomics, Center for Evolution and Medicine, School of Life Sciences, Arizona State University, Tempe, AZ 85287-5001, USA; rafasfontenele@asu.edu (R.S.F.); tkringen@asu.edu (T.K.); simona.kraberger@asu.edu (S.K.); 3 Entomology and Nematology Department, University of Florida, Gainesville, FL 32611, USA; dorotalp@ufl.edu; 4 Division of Biomedical Informatics and Personalized Medicine, University of Colorado Anschutz Medical Campus, Aurora, CO 80045, USA; darcyj@colorado.edu; 5 Structural Biology Research Unit, Department of Integrative Biomedical Sciences, University of Cape Town, Observatory, Cape Town 7701, South Africa

**Keywords:** microvirus, CRESS DNA virus, cryoconite, glacier, viral metagenomics, Antarctica

## Abstract

Antarctic cryoconite holes, or small melt-holes in the surfaces of glaciers, create habitable oases for isolated microbial communities with tightly linked microbial population structures. Viruses may influence the dynamics of polar microbial communities, but the viromes of the Antarctic cryoconite holes have yet to be characterized. We characterize single-stranded DNA (ssDNA) viruses from three cryoconite holes in the Taylor Valley, Antarctica, using metagenomics. Half of the assembled metagenomes cluster with those in the viral family *Microviridae* (*n* = 7), and the rest with unclassified circular replication associated protein (Rep)-encoding single-stranded (CRESS) DNA viruses (*n* = 7). An additional 18 virus-like circular molecules encoding either a Rep, a capsid protein gene, or other unidentified but viral-like open reading frames were identified. The samples from which the genomes were identified show a strong gradient in microbial diversity and abundances, and the number of viral genomes detected in each sample mirror that gradient. Additionally, one of the CRESS genomes assembled here shares ~90% genome-wide pairwise identity with a virus identified from a freshwater pond on the McMurdo Ice Shelf (Antarctica). Otherwise, the similarity of these viruses to those previously identified is relatively low. Together, these patterns are consistent with the presence of a unique regional virome present in fresh water host populations of the McMurdo Dry Valley region.

## 1. Introduction

Viruses influence ecosystems worldwide in many ways such as by modulating microbial population size, diversity, and metabolic outputs [1,2,3,4,5]. Viruses are thought to play an important role in structuring polar freshwater bacterial communities, in part because the low nutrient availability of these environments leads to lower abundances of ciliates grazing on the bacteria [6,7,8]. However, polar viral genomic data makes up a very small portion of available databases, inhibiting inference about their biogeography, evolution, possible hosts, or other interactions [9].

**Figure 1:**
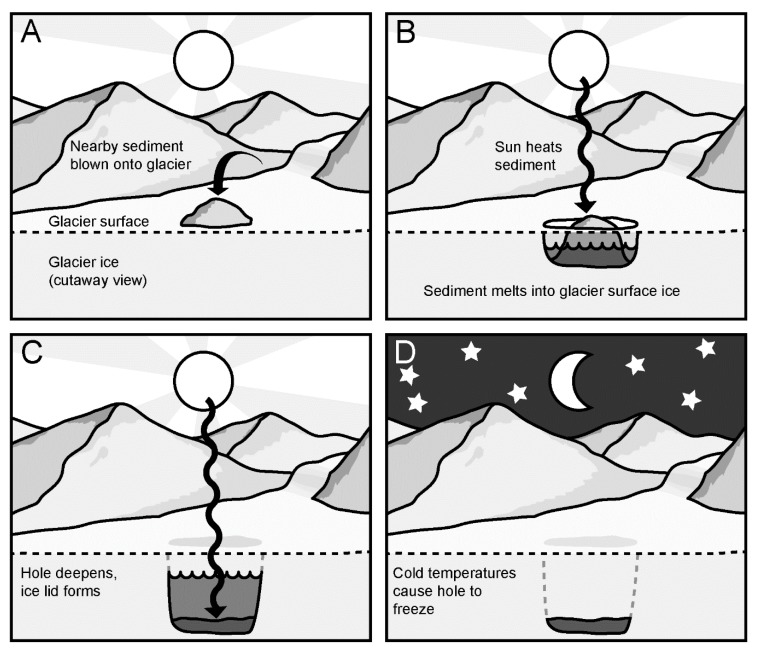
What is a cryoconite hole? (**A**): Cryoconite hole formation begins when sediment, often from nearby mountain slopes, is deposited onto a glacier’s surface. The sediment brings with it microbial life and nutrients, but it is dry and has low biomass. (**B**): The sediment has low albedo, meaning it absorbs solar radiation. This causes the sediment to warm, and melt into the glacier surface. This creates a relatively warm and wet environment; an oasis for microbial life. (**C**): Sediment melts deeper into the glacier until it is too deep to absorb enough radiation to continue melting. As temperatures get colder, an ice lid forms. (**D**): When solar radiation can no longer keep the hole warm and liquid, the water freezes and traps the sediment “puck” within the glacial ice. For Antarctic cryoconite holes, this freezing occurs at the end of the brief Antarctic summer, when there is no longer 24 h-sunlight to keep the hole warm.

Viruses may exert particular selection pressure and contribute to shaping the microbial communities of cryoconite holes [6], some of the most extreme polar freshwater ecosystems. Cryoconite holes (Figure 1) form when sediment deposited onto a glacier melts into the surface due to its lower albedo than the surrounding ice [10,11,12,13,14]. Melt continues until the sediment is too deep to absorb enough radiation to melt further, usually about 30–50 cm, at which point melt keeps approximate pace with ablation [13]. The sediment typically contains bacteria, algae, fungi, and microscopic animals such as tardigrades and bdelloid rotifers [10,15] that are active when the holes are melted [16], often becoming net photosynthetic communities [17]. Cryoconite holes can cover 0.1%–10% of the snow-free areas of glaciers and ice sheets [12,18,19], and contribute to regional carbon cycles [18] and nutrient fluxes [20]. These holes provide distinctive habitats for microbes characterized by temperatures near zero degrees, low light levels, and limited nutrient availability [21,22]. Their microbial community, while resembling that of streams near glaciers, has a distinct composition [23].

Viruses have been found in Arctic cryoconite holes, where phage infection rates and bacterial mortality rates appeared to be higher than those in temperate freshwaters [9,24,25]. The link between viral and microbial biomass in polar cryoconite holes has been suggested by a strong relationship between viral and bacterial abundances within Arctic cryoconite holes [24], and an increase in viral abundance following nutrient additions in microcosm experiments [26]. Virus genomes assembled from Arctic glaciers included novel groups of viruses, and predicted unusual host interactions, such as phages conferring immunity of their hosts to other phages using a CRISPR/Cas system encoded in the phage’s genome [27].

To our knowledge the viruses in Antarctic cryoconite holes have not yet been characterized. Cryoconite holes in Antarctica have been even more isolated over evolutionary time scales from distant temperate freshwater habitats by oceans than those in the Arctic. They often remain more isolated from one another over shorter time scales than Arctic cryoconite holes and other freshwater habitats, due to their ice lid (Figure 1). These active microbial ecosystems therefore experience a tight coupling of nutrient and community dynamics, in which viruses may play an important role.

High-throughput sequencing (HTS) technology has significantly increased the ability of researchers to detect and characterize viral genomes (viromes) in the past decade [28,29,30]. Yau and Seth-Pasricha [31] and Smeele, et al. [32] provide comprehensive reviews on our current knowledge of Antarctic virology. DNA viruses have been described from continental Antarctica [9] in soils [33,34,35], microbial mats [36,37], and lakes [38,39,40,41,42], and associated with various Antarctic animals [32,43,44,45] including fish [46,47]. Many of the viruses discovered in Antarctic aquatic ecosystems have single-stranded DNA (ssDNA) viral genomes [37,38,39]. Single-stranded DNA (ssDNA) viruses are currently classified into 13 families (*Anelloviridae, Bacillidnaviridae, Bidnaviridae, Circoviridae, Geminiviridae, Genomoviridae, Inoviridae, Microviridae, Nanoviridae, Parvoviridae, Pleolipoviridae, Smacoviridae* and *Spiraviridae*) and those that encode replication-associated protein (Rep) are known informally as circular Rep-encoding ssDNA (CRESS) viruses [48,49]. Once thought to be relatively rare, metagenomic studies have more recently revealed that ssDNA viruses in particular are ubiquitous in many environments and infect a diversity of hosts [37,50,51,52,53,54,55,56,57,58,59]. Families of ssDNA viruses, such as *Microviridae* comprise of bacteriophages, those that have been cultured infect enterobacteria and parasitic bacteria [60]. Weddell seals on the coast of Antarctica harbor ssDNA anelloviruses in their population [32,61,62], and members of *Microviridae* and unclassified CRESS viruses have been found in limno terrestrial environments of Antarctica [36,37,38,39]. 

Since no information is publicly available on viral communities associated with Antarctic cryoconite holes, here we applied viral metagenomic sequencing on samples collected in November 2016 from both the cryoconite holes and bare glacial ice of three glaciers in Taylor Valley to characterize viral genomes of ssDNA viruses. The communities of bacteria and microbial eukaryotes in cryoconite holes on these glaciers have greater species richness and biomass on glaciers near the coast (Commonwealth Glacier) than further inland (Taylor Glacier). Therefore, we expected to find a greater diversity of virus genomes close to rather than away from the coast. We also expected to find a greater diversity of virus genomes in the cryoconite holes with more diverse and active microbial communities than bare glacial ice which has much lower biomass. 

## 2. Materials and Methods 

### 2.1. Sample Collection

Taylor Valley is one of the McMurdo Dry Valleys, a largely ice-free region of approximately 4500 km^2^ on the western coast of the Ross Sea in Victoria Land, Antarctica [63]. The valley stretches approximately 40 km from the coast to where Taylor Glacier flows from the polar ice sheet [64]. Its landscape is comprised of ice-covered lakes and polar alpine glaciers between the exposed bedrock and large expanses of poorly developed soils [65]. A gradient of the biomass [66] and richness [22,67] of the bacteria and microbial eukaryotes inhabiting cryoconite holes in this valley corresponds with biogeochemical gradients in the surrounding soil [68]. The major wind patterns include stronger down-valley föhn winds that transport material from the ice sheet to the coast, and gentler up-valley winds that transport material from the coast [69,70].

We collected cores of frozen cryoconite holes and glacial ice from three glaciers spanning the length of Taylor Valley between the 7th and 17th of November, 2016: The Commonwealth Glacier, nearest the coast at the wide valley mouth, the Taylor Glacier, which defines the inland end of the valley approximately 26 km away, and the Canada Glacier, between the other two but adjacent to Commonwealth (only 6 km away) [64]. Both the cryoconite holes and bare glacial ice were sampled by drilling ice cores approximately 10 cm in diameter and 20 (±10) cm deep with a SIPRE corer. The cores were collected and stored in sterile Whirl-Pak bags (Nasco, USA). While the bare glacial ice cores were stored at –20 °C for six months and at –70 °C for another year before DNA extraction (see below), cores from the cryoconite holes were stored at –20 °C and processed within two weeks at Crary Laboratory at McMurdo Station (Antarctica). Sediment was melted at 4 °C for 24 h in acid-washed high-density polyethylene (HDPE) beakers covered with aluminum foil. The outer layer was first removed with Millipore water to prevent cross-contamination from the drill. The melted sediment was subsampled (0.3 g) for amplicon sequencing to profile communities of bacteria and microbial eukaryotes and measure biogeochemical parameters. Approximately 20 g was extracted for tardigrades and rotifers using a modified White tray method, following [66]. The sediments were placed on a tissue paper on top of a mesh screen at the surface of deionized water. After 24 h, the water was filtered through a 24 µm filter and concentrated in 5–15 mL deionized water. After 5–7 days of settling at 4 °C, the top 5–10 mL was examined under a dissecting microscope and actively moving ciliates, tardigrades, and rotifers were counted on a Zeiss Axiovert 35 Inverted Phase Contrast Microscope. Another 5 g of sediments were weighed, placed in a drying oven at 60 °C for 24 h, and re-weighed to calculate dry sediment equivalents. The remaining sediment was transferred to a new sterile bag and refrozen at –20 °C till processing for viruses.

### 2.2. Sample Processing and Nucleic Acid Extraction

**Figure 2:**
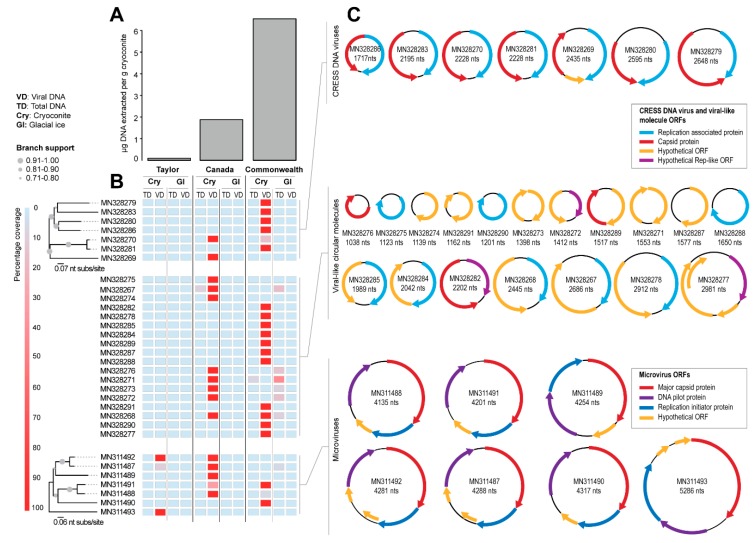
(**A**)**:** Total DNA concentrations extracted from cryoconite hole samples from the Taylor, Canada and Commonwealth glaciers for the amplicon sequencing performed in Sommers et al. [22]. (**B**)**:** Percentage of genome coverage of raw reads mapped from both total DNA (TD) and viral fraction (VD) from cryoconite and glacial ice to genomes recovered in this study with a Neighbor-joining phylogenetic trees showing genome-level relatedness. (**C**)**:** Genome/molecule organization for CRESS DNA viruses, circular molecules, and microviruses identified in this study. Hypothetical Rep-like ORF denotes sequences that either only have an endonuclease or helicase domain.

DNA extraction for amplicon sequencing of bacterial and eukaryotic communities was performed on 0.3 g cryoconite using the PowerSoil DNA Extraction Kit (MoBio, USA) and the concentration of DNA was measured with a Qubit fluorometer (Thermo Fisher, USA), both according to the manufacturers’ instructions. The characterization of the potential host communities (bacteria and eukaryotes) is described in Sommers et al. (2019). DNA concentrations were back-calculated to be per g cryoconite (wet mass), and are presented in Figure 2A as a proxy of biomass.

For viral genome analyses, the surfaces of the bare glacial ice cores were washed with sterile water. For each sample, 20 mL of melted material was filtered through a 0.2 µm filter. The filtrate containing the viral particles were then precipitated overnight at 4 °C with 15% PEG 8000 (*w*/*v*). The PEG precipitated solution was centrifuged at 14,000 rpm for 10 min and the pellet was resuspended in 400 µL of sterile water. To obtain the viral DNA (VD), 200 µL of resuspended solution was used to extract DNA from the viral particles using the High Pure Viral Nucleic Acid Kit (Roche Diagnostics, USA). Total DNA (TD) extraction from both cryoconite and glacial ice samples was performed by phenol:chloroform extraction protocol using 200 µL of melted sample material and processed as described in Di Pietro et al. [71]. Both the TD and purified VD of each sample were subsequently amplified using rolling circle amplification (RCA) with the TempliPhi™ kit (GE Healthcare, USA) for the enrichment of circular molecules [72,73]. The quantification of DNA was performed using the Qubit fluorometer (Thermo Fisher, USA) prior to library preparation. 

### 2.3. High-Throughput DNA Sequencing and Data Analysis

For each of the six samples (one from bare glacial ice and one from cryoconite hole sediment per each of three glaciers), both total DNA and purified viral DNA were individually sequenced on an Illumina HiSeq 4000 platform (2 × 100 bp paired-end libraries) at Macrogen Inc. (Korea). The quality-filtered raw reads were de novo assembled using SPAdes v 3.12.0 [74], and resulting contigs were analyzed by BLASTx [75] using a local viral protein database with all RefSeq protein sequences available in GenBank. Full-length genomes were identified for viruses belonging to the family *Microviridae* (*n* = 7) and the CRESS DNA virus group (*n* = 7). In addition, several viral-like (with hypothetical ORFs that have homology with viral-like sequences in GenBank) circular molecules (*n* = 18) were identified. 

All reads from each sample were mapped to each viral genome/circular molecule using BBmap [76] to determine percentage coverage across samples. The genome and circular molecule sequences have been deposited at GenBank under the accession numbers MN311489-MN311492 and MN328267-MN328291.

### 2.4. Sequence Similarity Network Analysis

A dataset of the major capsid protein (MCP) amino acid sequences of the microviruses was compiled based on data available in GenBank. A second dataset of the replication-associated protein (Rep) amino acid sequences was assembled from all CRESS DNA virus genomic sequences archived in GeneBank. For each dataset (MCP and Rep), using CD-HIT [77], we clustered sequences with a 90% sequence identity cut-off. Representative sequences from both data sets (MCP and Rep) were then combined with the Reps of the CRESS DNA viruses and the MCP of the microviruses identified in this study. These datasets were then used to construct Sequence Similarity Networks (SSN) using EST-EFI [78], with a minimum similarity score of 60 for the Rep and 200 for the MCP datasets. The resulting networks were visualized with Cytoscape V3.7.1 [79] with an organic layout.

### 2.5. Genome Characterization and Phylogenetic Analysis

The genomes of the microviruses identified were aligned with MUSCLE [80]. The same procedure was carried out for related CRESS DNA viruses identified in this study. To get an assessment of relatedness, especially for analysis of coverage among samples, each alignment was used to produce a Neighbor-joining phylogenetic tree with Jukes-Cantor substitution model and 1000 bootstrap iterations using MEGA5 [81]. Branches with bootstrap support <60% were collapsed using TreeGraph2 [82] and trees were midpoint rooted. 

Based on the SSN generated with the Rep amino acid sequence dataset, each cluster that contained an Antarctic CRESS DNA virus Rep with >2 sequences were separately aligned with MUSCLE [80] and the resulting alignment was used to infer a Maximum Likelihood phylogenetic tree using PhyML 3.0 [83], with the amino acid substitution model LG+G+I for all four clusters determined as the best-fit model by ProtTest [84]. Similarly, for the SSN analysis of the MCP amino acid sequence dataset, the only cluster containing Antarctic microvirus MCPs with >2 sequences was used to infer a Maximum Likelihood phylogenetic tree using the substitution model LG+G+I determined as the best-fit model by ProtTest [84]. All ML phylogenetic trees were midpoint rooted and branches with <80% approximate likelihood ratio test (aLRT) support were collapsed using TreeGraph2 [82]. 

BLASTp [75] analyses were undertaken for both the MCP and Reps to determine the closest related protein sequences available in GenBank, and between the encoded proteins from the genomes identified in this study. Using the best hit for each protein, pairwise amino acid identity was then determined using the program SDT v.1.2 [85].

## 3. Results and Discussion

Here we present the first description of ssDNA viruses from Antarctic cryoconite holes. Half (*n* = 7) of the full genomes assembled are members of a family which infects primarily bacteria, and the other half (*n* = 7) are unclassified CRESS DNA viruses, which typically infect eukaryotes. As expected due to their geographic isolation and extreme environment, their similarity to other characterized viral genomes is low overall. Furthermore, as would be expected from a pool of viruses actively infecting local hosts, the diversity of CRESS viruses mirrors ecological gradients in bacterial and eukaryotic diversity.

### 3.1. Identification of Viral Genomes

Seven genomes of CRESS DNA viruses (1717–2648 nts) encoding a Rep and capsid protein (CP) were identified (Figure 2B,C and Table 1). In addition, seven genomes of microviruses (4135–5286 nts) encoding at least a replication initiator protein, a DNA pilot protein, and a MCP (Figure 2B,C and Table 1) were assembled. Eighteen additional circular molecules (1038–2981 nts) encoding a Rep (*n* = 10), CP (*n* = 3), or hypothetical viral-like (*n* = 5) open reading frame (ORF) were also identified (Figure 2B,C and Table 1). We did not find any correlation of genome size with source of sample.

All of the circular molecules that were de novo assembled were from the cryoconite sediments and not the bare ice (Figure 2B). The greatest diversity of viral reads with a >50% match to the viral genomes and circular molecules identified in this study were found in the cryoconite collected from the Commonwealth Glacier, and the least diversity was in the cryoconite from the Taylor Glacier (Figure 2B). This pattern is consistent with larger biogeographic patterns of the cryoconite holes of the Taylor Valley, with cryoconite holes of the Commonwealth Glacier having greater amounts of DNA (Figure 2A), and greater abundance [66,86] and diversity [22,67,87] of bacteria and microbial eukaryotes. The pattern of viral diversity (viral genomes and circular molecules) in these sites mirroring potential hosts is therefore consistent with a pool of viruses able to infect local hosts.

One note of interest is that the viral reads were primarily detected in purified viral DNA but not in the total DNA (Figure 2B). This may be of methodological interest for future work on polar and other low-biomass virome work where bacterial and eukaryotic DNA may overpower the viral signal. In a survey of Arctic glaciers, a virus-to-bacterium ratio (VBR) was estimated at 13.6 for cryoconite and 7.5 for glacial ice [26], which is similar to or lower than ratios for Arctic lakes in the same study (VBR of 11–226). These ratios are higher than observations from the North Atlantic Ocean (VBR of 0.5–5), but lower than some from the Mediterranean Sea (VBR 0.3–138) [88]. Because the abundance of viruses does not scale linearly with the abundance of bacteria [89], oligotrophic systems with low bacterial abundance such as cryoconite holes would be expected to have greater VBR than more mesotrophic systems. Future work should include measures of viral abundance in Antarctic cryoconite holes, but no information is currently publicly available. However, the VBR in Antarctic freshwater lakes, including those of the Taylor Valley where we sampled cryoconite holes, have all been <10 [90,91], a commonly assumed ratio [89], making it plausible that ratios in Antarctic cryoconite holes may also be lower. 

### 3.2. Diversity and Relationships of CRESS Viruses and Viral-Like Circular Molecules

Within the Reps of the CRESS DNA viruses and Rep-encoding circular molecules, we identified the superfamily 3 (SF3) helicase and rolling circle replication (RCR) endonuclease domains (Table 2) that are conserved among the CRESS DNA virus Reps [48,49,92]. The RCR endonuclease domain consists of motif I, II, and III and is responsible for creating a nick at the origin of replication where rolling circle replication starts and ends. The SF3 helicase domain consists of motifs walker A, walker B, and motif C, and in some cases may contain an arginine finger [48].

**Figure 3:**
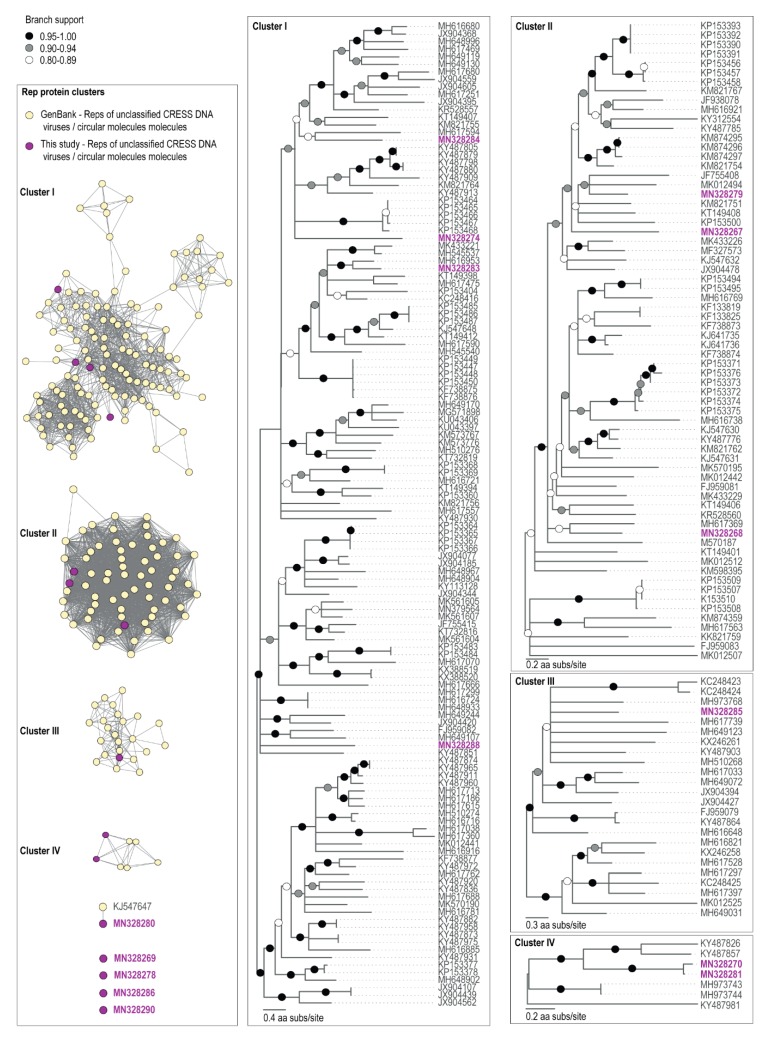
The sequence similarity network analysis of the Rep amino acid sequences encoded by viruses and viral-like circular molecules identified from cryoconite samples together with those of unclassified CRESS viruses from GenBank are shown in the left panel. Maximum Likelihood phylogenetic trees of Rep amino acid sequences of cryoconite viruses with representative Rep sequences of unclassified CRESS DNA viruses are shown to the right of the sequence similarity network analysis panel. The phylogenetic trees are rooted with representative Rep sequences of the unclassified CRESS DNA group. Branches with <0.8 approximate likelihood ratio test (aLRT) support have been collapsed.

None of the amino acid sequences of the Reps clustered with those of established ssDNA viral families (Figure 3). The closest BLAST matches within GenBank archived sequences of genomes assembled here were unclassified CRESS DNA viruses, with the exception of the Antarctic circular molecule (ACM) COCH21_215 (MN328290), a Rep encoding circular molecule of 1201 nts identified from the Commonwealth glacier cryoconite sample, whose nearest BLAST match was a nanovirus Rep (KX534391) sharing 38% amino acid identity (Table 3). Nonetheless, ACM-COCH21_215 is certainly not a nanovirus as it does not cluster with the Reps of nanoviruses (Supplementary Figure S1) which all share >53% amino acid identity. CRESS DNA viruses which currently do not fall into known families also dominate the fraction of ssDNA viral genomes found in Antarctic lakes [39].

The Antarctic virus (AV) COCH21_5 (MN328280) that we identified in the Commonwealth Glacier cryoconite sample encodes a Rep that has a 94% similarity (Table 3) to the Rep of McMurdo Ice Shelf pond-associated circular DNA virus-2 (KJ547647) that was identified from a freshwater pond approximately 70 km away, sampled in 1988 on the McMurdo Ice Shelf (Antarctica) [37]. At a genomic level, these viral genomes have similar genome organization with bidirectionally organized ORFs, and share ~90% genome-wide pairwise identity. Thus, it is highly likely that the pond and cryoconite sample support similar hosts for these viruses.

Besides the similarity to the coastal pond viral genome, none of the CRESS genomes assembled here had greater than 66% amino acid pairwise identity of putative Rep proteins with other viral genomes in GenBank (Table 3). Other studies of viral genomes from Antarctic freshwater ecosystems have found similarly unique ssDNA viral genomes when compared with genome databases [37,38,40], within which polar environments are underrepresented [9]. 

We note that AV-CAA_003_54 (MN328270) and AV-COCH21_74 (MN328281), identified from the Canada and Commonwealth glaciers respectively, encode Reps that share 94% amino acid identity (Table 3). They have similar genomes (~91% identity) with bidirectionally organized ORFs (Figure 2C). It is likely that these two viruses infect similar hosts. Those two glaciers are approximately 6 km apart, and both are on the coastal side of the dividing geologic feature of the valley, the Neussbaum Riegel [64]. Similarly, we note that there are some circular molecules (ACM-CAA_003_32, MN328267; ACM-CAA_003_40, MN328268; ACM-CAA_003_V_97, MN328271; ACM-CAA_003_V_107, MN328272; ACM-CAA_003_V_115, MN328273) that are present in Canada cryoconite and Commonwealth glacier ice samples, but absent in the cryoconite or ice sample from Taylor Glacier (Figure 2). The Taylor Glacier has lower biomass in its cryoconite holes (Figure 2A), and is approximately 20 km from the Canada Glacier (26 km from the Commonwealth Glacier) on the far side of the Neussbaum Riegel [64]. Tardigrades and rotifers are significantly less abundant in cryoconite holes on the Taylor Glacier than he Commonwealth and Canada Glaciers [66] in general, and the samples we sequenced reflected that trend for the total numbers of microinvertebrates. The cryoconite hole from the Commonwealth Glacier contained 9.0 tardigrades and 13.5 rotifers per g dry sediment (22.5 total), the hole from the Canada Glacier contained 3.6 tardigrades and 5.5 rotifers per g dry sediment (9.1 total), and the hole from the Taylor Glacier contained 0 tardigrades and 6.0 rotifers per g dry sediment (6.0 total). Based on the molecular metabarcoding of cryoconite samples from these same glaciers, algae and cyanobacteria follow patterns similar to those of tardigrades and rotifers [22].

### 3.3. Microvirus Diversity

Microviruses are known to infect bacteria, primarily those enterobacterial and parasitic bacterial included in well-studied systems such as human and livestock microbiomes [93]. All of the MCP amino acid sequences analyzed here cluster with the microvirus subfamily *Gokushovirinae*. Members of this subfamily (that have been cultured) have been found to infect obligate intracellular bacterial parasites such as *Bdelloivibrio*, *Chlamydia,* and *Spiroplasma* [94], and are speculated to infect Gram-positive bacteria in the human gut [95]. Nonetheless, they have been identified in various ecosystems and animal samples [51,57,58,93,95,96]. Their presence here could be related to the microbiomes of gut microinvertebrates such as bdelloid rotifers and tardigrades, as they have been found associated with invertebrates [54,96], or they could be infecting some other group of bacteria. 

**Figure 4:**
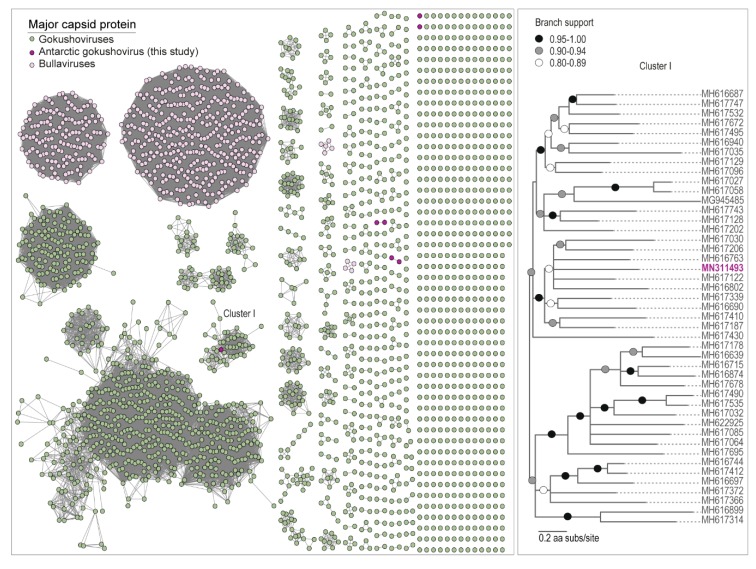
The sequence similarity network analysis of the major capsid protein (MCP) amino acid sequences of microviruses identified from cryoconite together with those available in GenBank is shown in the left panel. The Maximum Likelihood phylogenetic tree of MCP amino acid sequences for the cluster of MCPs of viruses from the large cluster (Cluster I) is shown in the right panel. Branches with <0.8 aLRT support have been collapsed.

Unlike the CRESS DNA viruses and viral-like circular molecules, we identified microviruses in samples from all three glaciers, with the greatest diversity from the Canada Glacier cryoconite holes (Figure 2B). The Antarctic microviruses (AMV) TYR_006_V_25 (MN311492) and AMV-CAA_003_V_1 (MN311487) were both identified in the Taylor and Canada glacier cryoconite samples, and have a very similar genome organization, and share ~88% pairwise identity. Additionally, a small portion of the reads (~10% genome coverage) from the Commonwealth and Taylor glaciers cryoconite sample map to AMV-CAA_003_V_1 (MN311487), which was identified from the Canada Glacier (Figure 2B). Both AMV-CAA_003_V_4 (MN311488) and AMV-COCH21_V_SP_16 (MN311491) were identified in the Canada and Commonwealth glacier cryoconite samples, and have a similar genome organization and share ~81% pairwise identity. Microviruses have previously been found in Antarctic lakes [38,39] and microbial mats [36]. Nevertheless, the MCPs of all the microviruses identified in this study share <59% pairwise amino acid identity with those of published microvirus genomes (Table 4). In the MCP amino acid SSN analysis, only one (AMV-TYR_006_V_SP_13; MN311493) formed a cluster with other MCPs of microviruses, four formed two pairs, and two are singletons (Figure 4).

## 4. Concluding Remarks

Here we confirm the presence of ssDNA viral genomes in Antarctic cryoconite holes capable of infecting diverse hosts. These genomes include both CRESS viruses (likely infecting eukaryotic cells) and microviruses that infect bacteria. In addition, the 18 viral-like circular molecules identified that did not appear to form a complete viral genome could be subgenomic molecules or components of multicomponent viruses like those observed for nanoviruses and unclassified CRESS DNA viruses [54,97]. The viral genomes presented here are not closely related to viruses from other regions of the world but bear some relation to one another and Antarctic viral genomes from nearby similar environments. Neither the likely eukaryotic-infecting CRESS viruses nor the bacterial-infecting microviruses that cluster with the subfamily *Gokushovirinae* had greater than 66% amino acid pairwise identity with proteins encoded by viral genomes deposited in GenBank. The one exception was a CRESS virus with 94% Rep amino acid pairwise identity to a virus from a coastal pond in the McMurdo Sound, which was sampled about 70 km away in 1988 [37]. However, two CRESS viral genomes identified here from two adjacent glaciers (Canada and Commonwealth glaciers) also had 94% Rep pairwise amino acid identity, suggesting that they likely infect similar hosts. Two microviruses were both identified in the Taylor and Canada glacier cryoconite samples, and they have a very similar genome organization and share ~88% nucleotide pairwise identity. Another pair of microviruses with similar genome organization and 81% nucleotide pairwise identity were identified from the Canada and Commonwealth glaciers. These patterns could be consistent with the presence of a viral community actively infecting both bacterial and eukaryotic hosts that has evolved in isolation from viruses outside Antarctica. This community may be a reflection of sources of cryoconite, or a reflection of active microbial communities within the cryoconite holes themselves.

Further evidence suggestive of an active pool of viruses in the region is that within the Taylor Valley, the pattern of cryoconite CRESS viral diversity mirrored those of bacteria and eukaryotes across the landscape, with the greatest diversity of viruses near the coast and the least diversity near the polar ice sheet. Microviruses, on the other hand, were found on all three glaciers, with the greatest diversity on the Canada Glacier. As expected, the viruses were identified from cryoconite hole samples, but not detected in lower biomass bare glacial ice.

These data and previous work on limno-terrestrial polar viromes suggest that there is much more to learn about the diversity and ecological roles of viruses in Antarctica [9]. Major gaps that future research should seek to address include basic goals such as defining the taxonomy and biogeography of Antarctic viruses [9]. To understand the role of viruses in polar ecology and evolution, it will be important to identify hosts, including host specificity and their impacts on those hosts, including their role in manipulating cellular function (e.g., through auxiliary metabolic genes) and moving genes between species, including genetic movement between species of viruses [7].

## Figures and Tables

**Table 1:** Summary of viruses and circular molecules identified in this study and their GenBank accession numbers.

Virus Group	Name	Accession
**CRESS DNA viruses**	Antarctic virus CAA 003 44	MN328269
	Antarctic virus CAA 003 54	MN328270
	Antarctic virus COCH21 47	MN328279
	Antarctic virus COCH21 51	MN328280
	Antarctic virus COCH21 74	MN328281
	Antarctic virus COCH21 78	MN328283
	Antarctic virus COCH21 111	MN328286
**Microviruses**	Antarctic microvirus CAA 003 V 1	MN311487
	Antarctic microvirus CAA 003 V 4	MN311488
	Antarctic microvirus CAA 003 V 9	MN311489
	Antarctic microvirus COCH21 V SP 16	MN311491
	Antarctic microvirus COCH21 V SP 13	MN311490
	Antarctic microvirus TYR 006 V 25	MN311492
	Antarctic microvirus TYR 006 V SP 13	MN311493
**Circular molecules**	Antarctic circular molecule CAA 003 32	MN328267
	Antarctic circular molecule CAA 003 40	MN328268
	Antarctic circular molecule CAA 003 97	MN328271
	Antarctic circular molecule CAA 003 107	MN328272
	Antarctic circular molecule CAA 003 115	MN328273
	Antarctic circular molecule CAA 003 147	MN328274
	Antarctic circular molecule CAA 003 151	MN328275
	Antarctic circular molecule CAA 003 179	MN328276
	Antarctic circular molecule COCH21 37	MN328277
	Antarctic circular molecule COCH21 39	MN328278
	Antarctic circular molecule COCH21 77	MN328282
	Antarctic circular molecule COCH21 94	MN328284
	Antarctic circular molecule COCH21 102	MN328285
	Antarctic circular molecule COCH21 141	MN328287
	Antarctic circular molecule COCH21 149	MN328288
	Antarctic circular molecule COCH21 162	MN328289
	Antarctic circular molecule COCH21 215	MN328290
	Antarctic circular molecule COCH21 226	MN328291

**Table 2:** Summary of the rolling circle replication (RCR) motifs of the endonuclease and SF3 helicase domains of the Reps encoded by viruses and circular molecules identified in this study.

Accession Number	Name	Endonuclease Domain			SF3 Helicase Domain			
		Motif I	Motif II	Motif III	Walker A	Walker B	Motif C	Arg Finger
MN328269	Antarctic virus CAA 003 44	FLTWPK	IHYHVC	GAVGYTGK	GVGFHGKSKFGE	VFDYE	VFAN	MSEDRW
MN328270	Antarctic virus CAA 003 54	LLTFAQ	AHFHAV	RAVEYVAK	GPSRYGKTVLAR	VLDDL	VLTN	
MN328279	Antarctic virus COCH21 47	ILTIPA	VHWQLL	AAEEYCGK	GVTGTGKSRTAW	VIDEF	ITSN	ALMRRL
MN328283	Antarctic virus COCH21 78	CFTWNN	PHLQGY	QNDRYCRK	GDSGCGKTRSVN	LVDDV	VTSQ	ALLRRF
MN328281	Antarctic virus COCH21 74	LLTFAQ	AHFHAV	RAVEYVAK	GPSRYGKTVLAR	VLDDL	VLTN	
MN328280	Antarctic virus COCH21 51	CVTIHI	IHWQMY	LAIEYCKK	GRSGLGKTQFAI	IFDDM	FTSN	AIRRRC
MN328286	Antarctic virus COCH21 111	AWTIYG	LHYQGQ	GSELYCQK	PDGNAGKTCFAK	IVDVK	VFSN	LSKDRW
MN328267	Antarctic circular molecule CAA 003 32	IATMPH	LHWQFV	AAIAYVWK	GRTGTGKSRRAW	VIDEF	ITSN	AFLRRL
MN328268	Antarctic circular molecule CAA 003 40	LLTIRQ	VHWQVL	AADEYVWK	GATGTGKSRLAW	VLDEF	ITSN	ALLRRM
MN328274	Antarctic circular molecule CAA 003 147	CYTLNN	PHLQGY	QNVTYCSK	GPPGTGKSRKAR	IIDDI	VTSN	AIQRRY
MN328278	Antarctic circular molecule COCH21 39	VFTKHF	IHWQGY	EAREYCMK	TIGGKGKTRLAT	IFDIS	FFSN	LSLDRV
MN328284	Antarctic circular molecule COCH21 94	CFTLNN	PHLQGF	QNRDYCIK	GQTGCGKTRSAT	IIDDF	VTSQ	AIMRRV
MN328285	Antarctic circular molecule COCH21 102	ALTFWD	IHYQSY	ENIAYCSK	GPTGVGKSHQAF	FNDFR	VTSS	QLLRRF
MN328288	Antarctic circular molecule COCH21 149	MWTLNN	PHLQGA	EALDYCVK	GPTGTGKSRSVL	FIDDF	ITSN	PLHRRF
MN328290	Antarctic circular molecule COCH21 215	CFTWNN	PHYQGY	EAIAYCTK	SQGNAGKTTFTK	VFDIN	VFSN	LSVDRL

**Table 3:** Pairwise amino acid sequence identities of the Reps and capsid proteins (CPs) encoded by viruses and circular molecules identified in this study with each nearest match within this dataset, and those of viral sequences in GenBank. The pairwise identities were determined using SDT v1.2 [85].

Name	Accession Number	Rep				CP			
		Nearest from Dataset		Nearest from GenBank		Nearest from Dataset		Nearest from GenBank	
		Sequence ID	Pairwise ID	Sequence ID	Pairwise ID	Sequence ID	Pairwise ID	Sequence ID	Pairwise ID
Antarctic virus CAA_003 44	MN328269	MN328290	26%	KF738885	31%	MN328276	47%	MH552476	31%
Antarctic virus CAA 003 54	MN328270	MN328281	94%	KY487857	50%	MN328281	87%	KY487857	52%
Antarctic virus COCH21 47	MN328279	MN328268	55%	KM821754	61%	MN328286	29%	MH552476	30%
Antarctic virus COCH21 5	MN328280	MN328268	30%	KJ547647	94%	MN328281	21%	KJ547647	78%
Antarctic virus COCH21 74	MN328281	MN328268	26%	KY487857	49%	MN328270	87%	KY487857	50%
Antarctic virus COCH21 78	MN328283	MN328284	40%	MH616953	66%	MN328276	23%	KY487851	25%
Antarctic virus COCH21 111	MN328286	MN328278	31%	MH617452	41%	MN328279	29%	KY487835	35%
Antarctic circular molecule CAA 003 32	MN328267	MN328268	55%	KM821754	61%				
Antarctic circular molecule CAA 003 40	MN328268	MN328267	55%	KM874297	63%				
Antarctic circular molecule CAA 003 147	MN328274	MN328284	41%	JX904420	46%				
Antarctic circular molecule COCH21 39	MN328278	MN328286	31%	MH616877	41%				
Antarctic circular molecule COCH21 94	MN328284	MN328288	42%	JX904420	45%				
Antarctic circular molecule COCH21 102	MN328285	MN328274	33%	KY487903	48%				
Antarctic circular molecule COCH21 149	MN328288	MN328284	42%	MH648933	45%				
Antarctic circular molecule COCH21 215	MN328290	MN328286	31%	KX534391	38%				
Antarctic circular molecule CAA 003 179	MN328276					MN328269	47%	MH552476	28%
Antarctic circular molecule COCH21 77	MN328282					MN328276	27%	MK012465	27%
Antarctic circular molecule COCH21 162	MN328289					MN328269	27%	MH616939	31%

**Table 4:** Pairwise amino acid sequence identities of the major capsid proteins (MCPs) of microviruses identified in this study, with each nearest match within this dataset, and those of viral sequences in GenBank. The pairwise identities were determined using SDT v1.2 [85].

Name	Accession Number	Nearest Neighbor within Dataset		Nearest Neighbor within Database	
		Sequence	Pairwise %	Sequence	Pairwise %
Antarctic microvirus CAA 003 V 1	MN311487	MN311491	56%	MK765582	59%
Antarctic microvirus CAA 003 V 4	MN311488	MN311491	90%	MH617700	61%
Antarctic microvirus CAA 003 V 9	MN311489	MN311492	53%	MK765646	54%
Antarctic microvirus COCH21 V SP 13	MN311490	MN311489	51%	MH617350	60%
Antarctic microvirus COCH21 V SP 16	MN311491	MN311488	90%	MH617700	61%
Antarctic microvirus TYR 006 V 25	MN311492	MN311487	92%	MK765582	59%
Antarctic microvirus TYR 006 V SP 13	MN311493	MN311492	29%	MH617122	64%

## Supplementary Materials

The following are available online at https://www.mdpi.com/1999-4915/11/11/1022/s1, Figure S1: Sequence similarity network analysis of the Rep amino acid sequences encoded by viruses and viral-like circular molecules identified from cryoconite samples together with those of classified and unclassified CRESS viruses from GenBank.
