# Supplementary material for: Single-Stranded DNA Viruses in Antarctic Cryoconite Holes
Source: Viruses. 2019 Nov 4;11(11):1022. doi: 10.3390/v11111022 (PMC6893807; doi:10.3390/v11111022)
Supplement: Supplementary file 1 [file viruses-11-01022-s001.pdf]

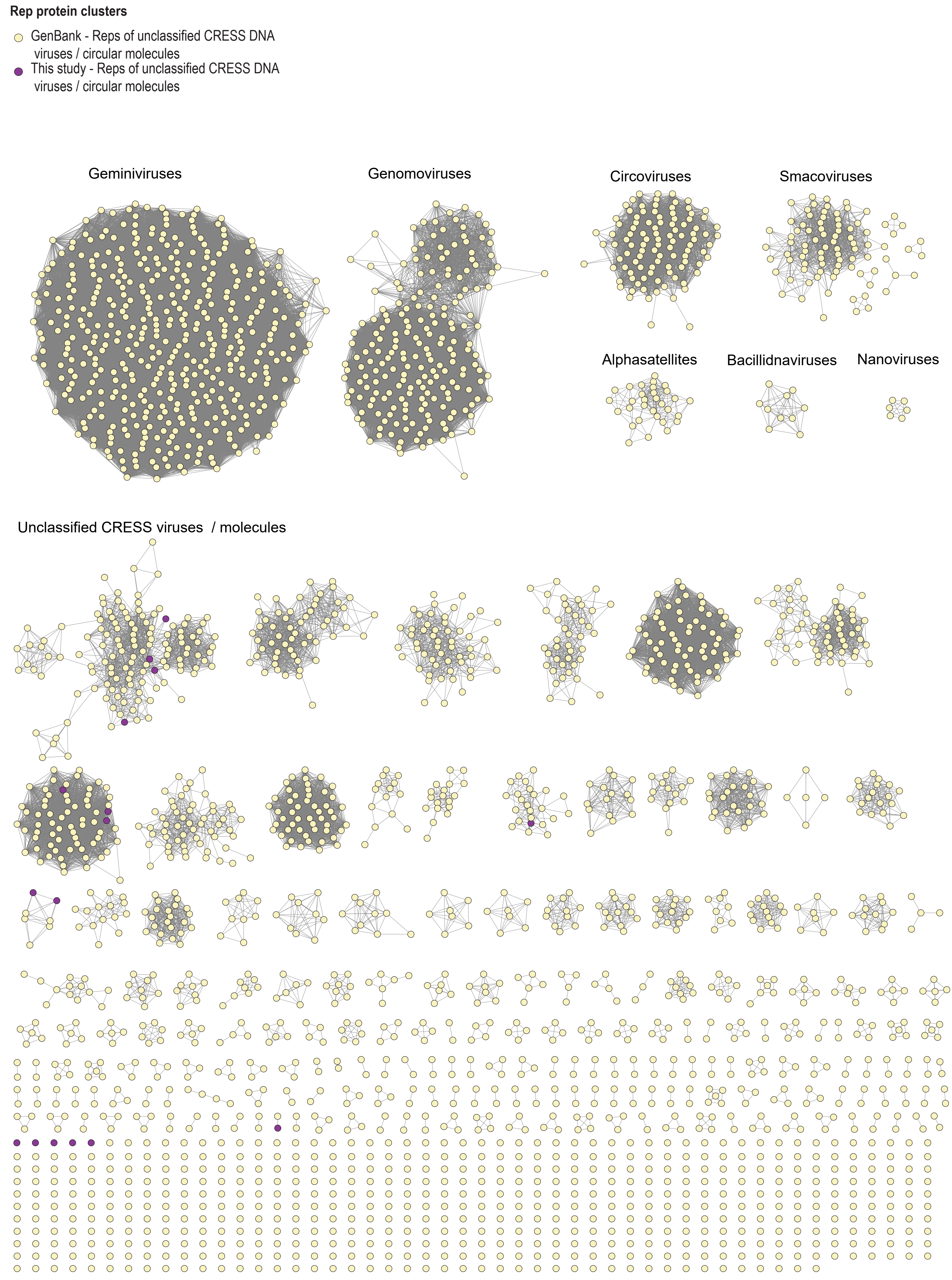

Supplementary Figure 1: Sequence similarity network analysis of the Rep amino acid sequences encoded by viruses and viral-like circular molecules identified from cryoconite samples together with those of classified and unclassified CRESS viruses from GenBank.
